# Perceptions of Physical Activity Participation Among Adolescents with Autism Spectrum Disorders: A Conceptual Model of Conditional Participation

**DOI:** 10.1007/s10803-017-3436-2

**Published:** 2017-12-13

**Authors:** Susann Arnell, Kajsa Jerlinder, Lars-Olov Lundqvist

**Affiliations:** 1 Faculty of Medicine and Health, University Health Care Research Center, Örebro University, P. O. Box 1613, 701 16 Örebro, Sweden; 2 School of Health Sciences, The Swedish Institute for Disability Research (SIDR), Örebro University, Örebro, Sweden; 3 Faculty of Health and Occupational Studies, University of Gävle, Gävle, Sweden

**Keywords:** Autism spectrum disorder, Adolescent, Physical activity, Participation

## Abstract

Adolescents with an autism spectrum disorder (ASD) are less physically active compared to typically developing peers. The reasons for not being physically active are complex and depend on several factors, which have not been comprehensively described from the adolescent’s perspective. Therefore, the aim was to describe how adolescents with an ASD perceive, experience and reflect on their participation in physical activity. Interviews with 24 adolescents diagnosed with high-functioning ASD, aged 12–16 years, were analysed with qualitative content analysis with an inductive approach. They expressed a variety of reasons determining their willingness to participate, which were conceptualized as: *Conditional participation in physical activities*. The present study presents an alternative perspective on participation in physical activity, with impact on intervention design.

## Introduction

Adolescents with an autism spectrum disorder (ASD) are less physically fit (Borremans et al. 2010), less physically active (Jones et al. 2017) and participate in fewer physical activities compared to typically developing peers (McCoy et al. 2016; Memari et al. 2015; Potvin et al. 2013). Few of these adolescents meet the physical activity (PA) guidelines of 60 min of moderate-to-vigorous PA each day (Bandini et al. 2013; Pan and Frey 2006), which leads to impaired health (Biddle and Asare 2011; Janssen and Leblanc 2010; Strong et al. 2005). Reasons for adolescents with an ASD not being physically active are complex and depend on several factors (Obrusnikova and Cavalier 2011). The most common reasons relate to intrinsic factors such as lack of motivation (Obrusnikova and Miccinello 2012; Stanish et al. 2015), low interest in PA (Obrusnikova and Cavalier 2011), low perceived motor skill competence (Loprinzi et al. 2015) and low enjoyment of PA (Eversole et al. 2016). Other reasons relate to the symptoms of ASD, such as impaired communication, limited social interaction and motor difficulties (Damme et al. 2015). In particular, their participation is hampered by their difficulties complying with others’ requests or adjusting to rules, regulations and social norms (Ostfeld-Etzion et al. 2016; Ghaziuddin 2002). This may be most apparent in the traditional contexts for PA such as physical education (PE) and organized sports (Ayvazoglu et al. 2015; Menear and Neumeier 2015). In these contexts, participation often requires both social interaction and athletic skills, which can be problematic for adolescents with an ASD.

Research on the benefits of specific PA interventions among adolescents with an ASD has shown improvements in behaviour, health and motor skills (Bremer et al. 2016; Dillon et al. 2016; Lang et al. 2010; Sorensen and Zarrett 2014; Sowa and Meulenbroek 2012). The feelings of adolescents with an ASD about PA have been investigated through questionnaires and compared to typically developing adolescents, revealing both similarities, for example the enjoyment of PA and awareness of PA being “good for them”, and differences, such as lower enjoyment of group PA among adolescents with ASD compared to typically developing adolescents (Stanish et al. 2015). Other studies have found that these adolescents face challenges regarding perceived physical ability, sensory issues and concerns about negative social interaction such as bullying or being compared to others when they participate in PE (Healy et al. 2013) and in leisure activities (Brewster and Coleyshaw 2011), which affects their preferences and choice of activities. Despite an awareness among professionals of the benefits of PA and of factors that may affect participation in specific activities, interventions have yet not fully succeeded in changing habits to increase PA levels among adolescents with an ASD (McCoy et al. 2016; Obrusnikova and Cavalier 2011; Pan et al. 2016; Stanish et al. 2015; Tyler et al. 2014). We may therefore assume that other aspects not accounted for in previous research are influencing participation in PA. Thus, there is a need for research that more comprehensively describes these adolescents’ views of participating in PA.

The aim of this study was thus to describe how adolescents with an ASD perceive, experience and reflect on their participation in PA.

## Method

### Participants

All adolescents aged 12–16 years diagnosed with an ASD (DSM 5 299.00) without a co-occurring intellectual disability (N = 190), registered at the Child and Youth Habilitation Centre in a county in the central region of Sweden, were asked to participate in this study. A letter of invitation with information about the study was sent to parents or legal guardians of the adolescent during a period from March to April 2015. An age-appropriate version of the written information was also sent to each adolescent. A follow-up phone call was made in order to answer any questions about the study. The adolescent and parents (or guardian) submitted written informed consent before participating in this study. Participants were assured that their identities would remain anonymous in all reporting of the study and that their personal information would be kept confidential. The study was approved by the Regional Ethical Review Board in Uppsala, Sweden (Approval No.: dnr 2014/243).

Twenty-four adolescents with a formal diagnosis of ASD agreed to participate in this study. The study included 17 boys and 7 girls, from grades 6–9 and aged 12–16 years. All adolescents followed the national curriculum of Swedish mainstream compulsory schooling. Thirteen adolescents participated in mainstream classroom education and 11 had an adapted form of schooling, including small group education or education at home.

As shown in Table 1, the adolescents’ PA habits and preferences varied greatly. Some were highly physically active and interested in sports, whereas others preferred being sedentary and lacked any interest in PA. For some, mandatory PE was their main context for being physically active. Among those who were physically active, the preferred PA varied: some preferred solitary activities whereas others enjoyed being part of a team. The choice of PA companions also varied: parents and siblings were commonly mentioned, but sometimes a friend was preferred. In summary, the adolescents in the present study form a heterogeneous group concerning their current PA habits and preferences.

**Table 1:** Attendee’s reported PA habits

#	Gender	PE	Leisure time		Social context
		Participation	Type of PA	PA frequency (per week)	
1	B	Excluded	Team sport, gym	3–5	T, F, P
2	G	Regular	Running, horseback riding, swimming	1–2	F, S, e/c
3	B	Regular	Gym	3–4	S, R
4	G	Excluded	Walking, individual PA	< 1	P
5	G	Excluded	Swimming, walking	1–2	P, R
6	G	Excluded	–	–	
7	B	Occasional	Swimming, cycling	1–3	P, S, R
8	B	Regular	Team sport	2	T, F
9	B	Regular	–	–	–
10	G	Regular	Gym, horseback riding	2–3	P, e/c
11	B	Regular	–	–	–
12	B	Regular	Running, individual PA	< 1	P,S
13	G	Excluded	Walking	< 1	–
14	B	Regular	–	–	–
15	G	Occasional	Running, individual PA	2–3	–
16	B	Regular	Team sport	2–3	T
17	B	Occasional	Water sport	2	F
18	B	Occasional	Individual PA, martial arts	2–3	–
19	B	Regular	Team sport, gym, dual PA	3–4	T, P, F
20	B	Regular	Walking	2–3	P
21	B	Regular	Team sport, cycling, running	3–4	P,S, F
22	B	Regular	–	–	–
23	B	Occasional	Cycling	< 1	S
24	B	Regular	Cycling, individual PA	3–4	F

Gender: B = boys, G = girls. Type of PA: team sport = football, floorball etc., individual PA = individual exercise in the home setting, dual PA = tennis, badminton etc. Social context: T = team, F = friends, P = parents, S = siblings, R = relatives, e/c = escort/contact person

### The Interviews

The adolescents’ perceptions, experiences and reflections concerning participation in PA were collected through qualitative interviews. An interview guide was developed for the study in order to cover the adolescents’ experiences in a range of PA contexts. To enhance the construct validity of the interview guide, four pilot interviews were performed prior to the interviews. Based on the pilot interviews the interview guide was revised and refined, allowing more context-specific and directed questions, in order to better capture the adolescents’ perspective of participation in PA in different contexts. The pilot interviews also gave insights into how to approach adolescents with an ASD in an interview situation with respect to the difficulties in social interaction and communication associated with these disorders. The four pilot interviews were not included in the results.

The individual interviews took place according to each adolescent’s preference: at the adolescent’s home (n = 10) or at their local habilitation centre (n = 14). Two interviews were conducted with a parent present in the room but not taking any active part. The first author, an experienced physiotherapist who had no professional connection with the adolescents, conducted all interviews to ensure internal consistency. Prior to the interviews, the adolescents were assured of confidentiality and reminded that they were free to stop their participation at any time without being obliged to give any explanation. The interviews lasted between 30 and 80 min. They were conducted as conversations and started with a discussion of the terminology used in PA contexts. The adolescents were asked to narrate their perceived experiences of PA and participating in PA in different settings. Follow-up questions were posed in order to encourage further and more reflective narrations or to clarify the adolescent’s answers. All interviews were digitally recorded with the participant’s permission and transcribed verbatim by the first author. The quotations that are presented in the results were professionally translated in order to capture the adolescent’s voices; the text may thus include grammatical errors found in transcribed speech, as well as jargon and slang phrases used by this population.

### Data Analysis

An inductive qualitative latent content analysis was used to analyse the material and to reveal the underlying meanings of the interview texts (Graneheim and Lundman 2004). Since qualitative content analysis aims at describing variations of the phenomenon under study, the focus was on finding similarities and differences in the text, not on how many of the adolescents expressed the same experiences. Initially, all the transcribed interviews were read through to obtain a sense of the overall content of the data. The authors then conducted the analysis in two phases. First, all authors developed a preliminary coding structure by selecting one of the most comprehensive interview transcripts. Each author read the transcript independently and identified any statement pertaining to PA. The statements, also referred to as meaning units, are constellations of words or sentences related to each other through the content and context (Graneheim and Lundman 2004). Thereafter, the meaning units were condensed and designated as codes by each author independently. The authors then met to compare and consolidate the codes. The first author completed the coding process for the remaining interview transcripts. All codes sharing a commonality were formed into tentative categories and themes, using the NVivo 10 software program (QSR International 2014). In the second phase, all authors met to discuss the tentative categories and themes. Differences of opinion were resolved via consensus until the final subthemes were identified, leading to one overarching theme.

## Results

The analysis resulted in a main theme with five subthemes that encapsulated the adolescents’ experience of participation in PA (Table 2). Statements from each adolescent are represented in almost all subthemes. In order to reflect the inductive analysis process, the subthemes that emerged are presented first and the main theme concludes this section. Representative quotations are used to illustrate the adolescents’ perceptions, experiences and reflections concerning participation in PA.

**Table 2:** The main theme, subthemes and categories

Categories	Subtheme	Theme
• Perceived competence in physical activity • Self-confidence • Vulnerability	Competence and confidence	Conditional participation—but it depends
• Enjoyment • Meaningfulness • Promotive participation • Self-regulation	Motivation	
• Adjustment to social demands • Adjustment to activity demands • Environmental demands	Adjustment to external demands	
• Familiarity • Being prepared • Knowing what to do	Predictability	
• Possibility to choose • Possibility to influence • Activity availability and accessibility	Freedom of choice	

### Competence and Confidence

The adolescents indicated in their narratives that they perceived their physical ability, competencies in PA and self-confidence as important factors that affected their willingness to engage in PA. Perceived low ability in PA and low self-confidence made them feel more vulnerable during PA. The adolescents expressed having both low and high competencies in PA. Their narratives also highlighted that different kinds of competencies were required for successful participation in PA, for instance motor skills and physical abilities combined with knowledge about the activity itself, i.e., knowing how to perform it.

It’s a tactical game, if everyone does what he has to do it’ll work…In fact, I have only done this for one year but most people tell me it looks as if I’ve been playing for three years... I have got the knack of the sport so damn easily and I have sort of developed quite quickly in it (boy 1).

The adolescents expressed that, in order to participate in PA at all, they had to have a certain minimum level of physical competence; if not, they were less likely to be willing to participate.

I sometimes have problems with the ‘motions’…I lack the automatic ability…to be able to improvise what motions to use, sometimes I must think beforehand which ones to use…it’s hard when I don’t get the flow that’s needed and even if I plan it, it doesn’t turn out right (boy 20).

On the other hand, adolescents also perceived themselves as competent in some specific type of PA, especially in activities that they considered easy and were used to.

As with competence, some adolescents felt confident whereas others expressed a lack of confidence during PA. Lack of confidence could be related to their performance in PA but also to low self-esteem related to their persona, body and appearance. This lack of confidence, particularly when related to their persona, led to feelings of insecurity: “I was ashamed and felt sick…I couldn’t cope…either I didn’t understand what they meant or I didn’t have the physical fitness required” (boy 9).

In addition, perceiving oneself as lacking in competence and being unconfident in PA led to a perceived vulnerability. Whereas some found that PA gave them a respite from stress, others said they became anxious and stressed out merely by thinking about physical activities. Particularly in conjunction with PE, feelings of anxiety and stress were reported, especially by the girls: “There was such a terrible lot of anxiety involved with PE…A whole day could be ruined just because of the PE…I almost didn’t want to go to school if we were supposed to have PE that day” (girl 15).

Vulnerability that was related to their body and appearance was particularly reflected in the changing room and in the showers at PE. Low perceived body satisfaction, due to physical changes associated with adolescence led to high levels of insecurity that affected the adolescents’ actions: “I don’t like exposing myself…I waited until everyone had been in the shower…was the only one still showering…I was wary of my body…it’s hardest in the changing room…then you might get comments” (boy 1).

### Motivation

According to the adolescents, the enjoyment and perceived meaningfulness of the activity were the two main aspects that influenced their motivation to be physically active. If PA was perceived as fun and important, and conducted with specific persons selected by the adolescents themselves, these were strong motivators for participation in PA. But, even where the activity was enjoyable and meaningful, the adolescents still reported having difficulties initiating PA themselves, indicating low self-regulation skills.

Both enjoyment and discomfort associated with being physically active were expressed. Enjoyment of the activity itself was a primal motivator for attendance and engagement in PA, and it increased their activity levels: “I don’t miss one single practice, I like it and I’m motivated…as long as it’s fun you want to do more and then you get more exercise” (boy 17). Having someone to be physically active with, who was undemanding and supportive, further increased their enjoyment, since participation in PA with someone specific was preferred to being completely solitary during PA.

The adolescents’ narratives further revealed that the perceived meaningfulness of the activity was another essential motivating factor, sometimes being crucial for involvement and engagement in PA. Health-enhancing PA and activities with a specific, useful purpose or goal were considered as meaningful by the adolescents. The motivation for participating in PA and in PE varied especially strongly according to this purpose or goal. Some adolescents referred to PE as unimportant, although they were aware of its purpose and the benefits of being physically active. Others participated in PE even though they did not find the activities meaningful or enjoyable, but only because it was required of them, and some attended only in order to receive a grade in PE.

I participate as much as I can [in PE]…but at the end of the lessons…I often get understimulated…I tend to keep away…to be more passive…don’t make an effort…don’t take any initiatives of my own…the activity is simply too boring…

To me personally it is not important at all [to take part in PE]…the reason for me to take part is that I don’t want to fail…if it wasn’t for that I wouldn’t participate (boy 20).

However, some of the adolescents were exempted or excluded from PE, whereas others only participated occasionally, depending on the type of activity during the PE class: ‘I have no PE in school, I’ve ruled it out…I thought it was unnecessary and it would be hell…but as long as it is theory, I’m in’ (girl 6). Where meaning was lacking, the physical activities were perceived as unnecessary and pointless, leading to an unwillingness to participate: ‘Football is useless…the only thing you learn from football is to run chasing after a ball, like a dog’ (boy 18).

The adolescents showed an awareness of the health benefits of regular PA. The health-promoting aspects were thus also a strong motivator in terms of gaining better health or a fitter body. The opposite, a fear of ill health and an avoidance of obesity, were thus also reported motivators of PA: “I run…want to keep fit, I don’t want to get fat…I care about my weight” (girl 10).

Despite their awareness of the physical and mental health benefits of PA, the adolescents described that they struggled with their own physical inactivity and self-regulation. Difficulties initiating and maintaining an activity were reasons for not being physically active: “Physical activities are important, of course, but…if I get the chance to do something else I’ll do something else…either I don’t have time or energy enough to fancy any activity…” (boy 9).

Their narratives also showed that PA and their physical and mental wellbeing had a reciprocal influence on their PA habits. Being physically active enhanced their wellbeing and self-esteem: “When I am not active I get sulky, irritated and insecure… I feel better from it [exercising]…it’s made me accept that I am doing all right” (boy 1). Equally, being aware of the positive health outcomes but not being physically active could lead to feelings of anguish: “I should activate myself more than I do…but lately I haven’t made it since I haven’t been feeling so well…so I have sort of put it aside…instead it gives me anxiety…a feeling that I am not active enough” (girl 4).

### Adjustment to External Demands

The adolescents reported that they experienced a variety of external demands during PA, such as social demands, demands regarding the activity itself and environmental demands, all affecting their participation in different ways.

They perceived social demands in conjunction with social interaction during PA. This was a reason for not participating, especially if it was in a social context they were uncomfortable with. More specifically, they described that they had difficulties adjusting to others, which lead to unwillingness to participate: “It is hard and tiring to have to adjust to what other people say and to have activities together, then I lose interest” (boy 20). For some adolescents, these demands led to reduced involvement in group activities or team sports and instead they increasingly preferred solitary PA. Social situations also evoked fear of spoiling the fun for others, which further contributed to unwillingness to participate in PA. Being watched and judged by others was another challenging aspect of PA, leading to anxiety about others’ opinions and comments and to fear of making a fool of oneself. Especially the adolescent girls expressed concerns regarding being different compared to their peers: “I don’t like being watched…it’s tough…I’m scared of being judged …I don’t want to be like others but I want to do it my own way without being judged” (girl 6).

But even being judged in a positive light could be troublesome, as the adolescents were ambivalent regarding encouragement, depending on who, where and in what situation the encouragement was given: “I can’t take encouragement, it disturbs me, I get unfocused and then it’s hard for me to concentrate and it gets embarrassing” (boy 3).

Many physical activities are competitive, particularly team sports and PE activities. For some adolescents the competitive element in the PA was a reason for not participating; for others it was a triggering factor that enhanced their willingness to participate: “I work harder, have more go when it is a competition, and the results are usually better since it’s fun to win” (boy 21). The perceived seriousness of the PA further affected their willingness to participate. Games and playful team sports that included PA were preferred by the adolescents to more serious team sports where specific performance requirements were more common.

The adolescents also reported that certain elements in the environment, such as insects, high temperatures and rainy or sunny weather, affected their willingness to participate in PA. Consequences of environmental factors perceived as negative could be a reason for reduced participation in PA: “I don’t like sweating,’cause then I have to take a shower…that’s one of the things that stops me from training” (girl 6). Other environmental factors, such as loud and noisy situations could inhibit their willingness to participate in PA: “Dancing is tiresome…too much noise…I can’t focus” (boy 18).

Nevertheless, many of the adolescents reported that they participated in PA even though it meant adjusting to different kinds of demands, but only if the demands were perceived to be on an acceptable level from the adolescent’s point of view. If the demands were perceived as overwhelming, willingness to participate subsided, whereas when adapted physical activities were offered, willingness to participate increased. The adolescents expressed frustration that a lack of physical activities adapted to their needs limited their ability to participate in PE: “The bad thing about PE is that it has been developed for those who already are active…and you shouldn’t be forced to worry about PE” (girl 15).

### Predictability

In order to feel comfortable about participation in PA the adolescents expressed a need for predictability of the activity itself and how they should perform it. Being familiar with the activity (both the type of activity and the setting), being prepared and knowing what to do increased a sense of predictability, which played an important role for the adolescents’ willingness to attend and engage in PA.

Performing familiar activities in a familiar setting was connected to positive experiences, thus enhancing the predictability of the activities, whereas unpredictability was a cause of insecurity: “I tend to lose confidence…it sort of depends on how many times I’ve done it and whether I had fun…then it usually gets easier, anyway” (girl 4).

A familiar social context was likewise reported as positive, in particular performing the activity with someone they knew and trusted: “It might help to have a friend by your side, someone who can motivate you…someone who knows your ways…someone who dares to push you” (boy 3).

Similarly, they wanted *to be prepared* for the activity. Knowing what to expect beforehand allowed them to plan their own participation and engagement in the activity. The adolescents found it important to know what to do and how to perform during the PA, since perceptions of uncertainty linked to the PA, such as not knowing what to do, or what expectations to have of their own performance, led to feelings of uncertainty. Not being prepared was perceived as stressful:

…You never knew what was happening…we were supposed to take out things, we were supposed to do this and that, afterwards we were told what it was [we were going to do] I didn’t like it at all…besides, I had difficulties with almost everything (boy 9).

Particularly the PE context and participating in PE was considered unpleasant due to insufficient information about the activities and insufficient instructions “…to explain in a better way how you should do it…demonstrate it…and make amendments for you…[since] you don’t ever want to say that you don’t understand” (girl 6).

### Freedom of Choice

The adolescents also emphasized the importance of freedom of choice and having an opportunity to influence the PA themselves: “you shouldn’t force anyone…you should be free to decide for yourself…now I am training’cause I want to [and] feel like it” (boy 17).

The adolescents wanted to make active *choices* regarding the activity itself, the activity context and the social context, which was expressed as a condition for willingness to participate at all. Since participation in PE is mandatory, it resulted in perceptions of reduced freedom of choice and thus reduced willingness to participate and a negative attitude toward PE: “[PE] limits the free choice of the individual…it tries to make everyone do all kind of things…which is no good” (boy 20).

The PE teacher had a cardinal role affecting the adolescents’ participation in PE by offering them possibilities to influence the choice of activities and PA companions during PE. According to the adolescents, it was important that the PE teacher was keen, responsive and clear, besides being understanding of their abilities and needs: “I have spoken to the PE teacher and we’ve decided that if there is something I don’t like then I don’t have to join in…more often than not I’m in… [refused] dancing and football” (boy 3). In order to cope with PE, the adolescents further emphasized the need to be allowed to select their activity partner. Not being able to freely choose PA companions from among their peers led to feelings of uncertainty: “I hate it when there are so many team activities, then you can’t choose who you want to work with and it gets scary” (girl 4). The adolescents thus clearly expressed a wish to both select and exclude different aspects of PE. Being offered a possibility to influence the activities was perceived as something positive since it affected the feeling of control.

Freedom of choice was limited by the activities that were *available and accessible*. According to the adolescents, preferred and age-appropriate activities were not always available, and sometimes a lack of transportation to the PA hindered them from participating: “I’d have been more physically active if there had been a beginners’ group for people my age…[but] there are no dancing beginners’ groups for young people my age” (girl 15).

### The Overarching Theme: Willing to Participate, but it depends …

The adolescents were a heterogeneous group in regard to their preferences, habits and needs, but a common theme was that some specific individual preferences had to be fulfilled. A common statement regarding their PA participation was, “but it depends on….”. This sentiment was found in all subthemes and was conceptualized as *Conditional participation*. Even where most conditions were met, such as being competent and confident in an activity, appreciating the activity itself, having had the opportunity to choose to attend and knowing how to perform it, the adolescent could choose to abstain simply because of a “wrong person” appearing during the activity.

The elements of conditional participation were found both during leisure time PA and during mandatory PE, but the PE stood out as an especially challenging context for the adolescents to cope with. During PE, the level of demands was perceived as high, which affected the adolescents’ willingness to participate. PE was highly regulated, with low responsiveness to the adolescents’ specific needs and thus did not allow for individual solutions. Consequently, the adolescents wished for a certain level of freedom of choice, another consistent finding in all the subthemes, whereby the perceived control was enhanced in an otherwise unpredictable PA context.

## Discussion

The aim of this study was to develop an understanding of the perceptions, experiences and reflections of adolescents with ASDs about participation in PA from their own perspective. According to the adolescents with an ASDs’ narratives, their experiences, PA habits and willingness to participate in PA varied markedly, showing heterogeneity within the group not fully captured in previous research.

Intrinsic factors such as confidence and competence were evident in the adolescents’ narratives as important for their willingness to participate in PA. This corresponds to research showing that higher levels of competence predict greater enjoyment and intensity of involvement in PA among children and adolescents with and without disabilities (King et al. 2013; Humbert et al. 2006). According to the adolescents in the present study, perceived enjoyment in its turn was crucial for motivation, especially for their willingness to attend and to get involved in PA.

Other aspects that affected the adolescents’ willingness to participate in PA were the different kind of external demands that the adolescents perceived. The perceived social demands were prominent, leading to increased levels of anxiety and uncertainty. One reason for increased anxiety was that social interaction during PA was experienced as stressful, which is in line with research showing that anxiety and fear in connection with peer relationships are common among adolescents with an ASD (Brewster and Coleyshaw 2011; Chen et al. 2016). Team sports were considered particularly challenging, since the activity itself requires both social interaction and a certain measure of social skills. In fact, less interaction with other participants can make activities more predictable, tolerable and thus even enjoyable by adolescents with an ASD (Ayvazoglu et al. 2015; Muller et al. 2008; Potvin et al. 2013; Stanish et al. 2015).

The adolescents also stressed that the predictability of the activity and the freedom to choose between activities were two crucial aspects affecting their willingness to take part in PA. Being prepared, knowing what, where, how and with whom to perform enhanced their willingness to participate, especially if they were familiar with these different aspects. This can be due to intolerance of uncertainty, which is related to anxiety and by adolescents with ASD reported reasons to restricted repetitive behaviour (Joyce et al. 2017). The present study also found that the desire for freedom of choice was a significant factor affecting their participation, and the PE context stood out as especially demanding.

PE is mandatory in schools and autonomy is reduced compared to PA in leisure time. PE often includes elements of evaluation of abilities and performance, which the adolescents in the study disliked. Despite their reluctance to participate in PE, many of the adolescents still participated in order to receive a grade in that school subject.

### Conditional Participation in PA

**Fig. 1:**
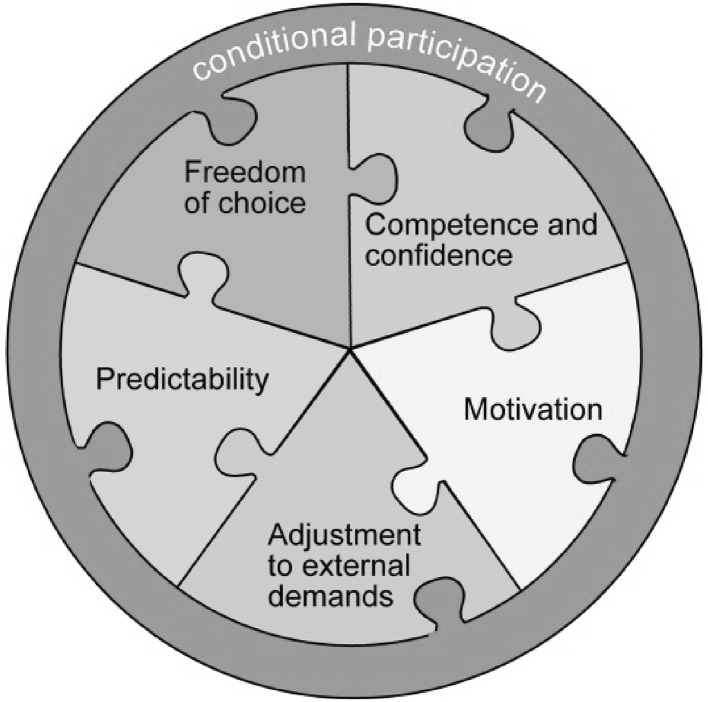
The conditional participation model

During the interviews, the adolescents frequently said, “but it depends” or equivalent expressions. This sentiment was found in all subthemes and was conceptualized as *Conditional participation*. The factors that the adolescents expressed were organized into a model of conditional participation, as outlined in Fig. 1. The model consists of five interdependent aspects (i.e., the subthemes) and proposes that, for sustainable participation in PA, individual conditions in all five aspects have to be met. With fewer conditions met, PA participation can still be present but will be less likely and less sustained. The model is conceptual and does not put any weight on the relative importance of each condition for willingness to participate. However, conditions regarding freedom of choice and perceptions of meaningful activities have previously been identified as influential for PA participation (Haegele and Sutherland 2015). This suggests that being able to choose and influence different aspects of participation with regard to when, where, with whom and to what extent, may be cardinal for increased participation in PA.

The conceptual model is based on the subthemes that emerged inductively from the interviews with adolescents with an ASD, yet it has similarities with models on participation among children and adolescents in general, such as Imms et al. (2017) “family of participation-related constructs” (fPRC). Factors affecting participation categorized in the fPRC framework (Imms et al. 2017) as “person-focused processes” and “environment-focused processes” were also found in the present study. “Person-focused processes” were expressed in terms of competence, confidence, motivation and being able to choose and influence physical activities, whereas “environmental-focused processes” included availability and accessibility of PA. However, the adolescents in the present study highlighted other concepts not described in the fPRC model, such as the need for *predictability, adjustment to external demands* and the demand of fulfilling specific individual conditions for participation to occur. This demonstrates that different processes may be salient among adolescents with an ASD compared with typically developing youth and youth with other disabilities. Common difficulties related to ASD, such as impaired social interaction skills, motor difficulties and intolerance of uncertainty, can to some extent be controlled by clearly expressed conditions regulating the PA participation.

### Limitations

While this study provides additional insight into the experiences and perceptions of adolescents with an ASD about participation in PA, some limitations and methodological considerations have to be addressed. First, the result is limited to those adolescents with high functioning autism who felt comfortable taking part in an interview, and there is no data from a comparative group of age- and gender-matched adolescents without ASD. Even though the sample included a range of adolescents with ASD, from those who were very physically active to those who were mostly sedentary, the descriptions of experiences of participation in PA may not fully reflect the source population in this study. Adolescents with more severe ASD impairments may experience other demands and may express other conditions, leading to the emergence of other categories and subthemes. Thus, it cannot be excluded that some new dimensions might emerge in the model with a more heterogeneous sample. It should be noted that the purpose of this study was not primarily to generate data that could be generalizable but rather to increase our understanding of how young people with an ASD experience and perceive participation in PA.

Second, only seven girls were interviewed compared to 17 boys. Although this corresponds to the epidemiology of the ASD population, the results may not reflect the girls’ experiences to the same extent as the boys’. Some gender differences were observed with regard to how challenging they found PA situations. With more girls, the study might have unveiled more gender differences.

Third, interviewing adolescents with an ASD can be difficult, since there is a risk that they might respond in a way that they think the interviewer wants rather than speaking out their own perceptions and feelings. In order to enhance their confidence in the interview situation the interviewer’s bias was discussed and the adolescents were informed that there were no right or wrong answers to the questions, focusing instead on the adolescent’s own experiences and perceptions of PA. The four pilot interviews that were conducted prior to the interviews were vital to refine the interview guide in order to better focus on the adolescents’ perspectives and narratives.

Finally, attention must be paid to the researchers’ preconceptions as a result of their professional background and experiences that affect the research (Malterud 2001). The first author is an experienced physiotherapist in child and adolescent habilitation with focus on adapted PA. The co-authors had a supervisory role and both are experienced researchers, one with deep knowledge in ASD and the other specialized in adapted PA. Thus, the authors represent different but complementary disciplinary perspectives, reducing the risk of unilateral or biased interpretations of the results.

### Clinical Implications and Future Research

Even though some of the attitudes expressed by the adolescent participants in this study are similar to those of adolescents without an ASD, their distinctive demands to participation in PA stood out. Future research is needed to examine whether and to what extent the adolescents’ demands regulate their PA participation. A survey, developed from the conditional participation model, could be used in a comparison of conditional PA participation in adolescents with an ASD and typically developing adolescents. The findings in this study suggest that challenges expressed by adolescents with an ASD can be misinterpreted as unwillingness to participate in PA, even though they in fact are willing to participate but refrain due to some individual condition not being met. One solution to promote PA among adolescents with an ASD is to teach them strategies for dealing with some of their conditions not being met and thus to enhance their “inner readiness” to meet perceived demands and challenges (Camargo et al. 2014).

Adolescents with an ASD may further lack equal opportunities with others to participate in PA, especially in PE, because of poor physical skills, or a perceived demand to adjust to specific activities and comply with requests, rules and social norms (Ostfeld-Etzion et al. 2016; Williams White et al. 2007). Failure to fully recognize the individual needs of adolescents with disabilities constitutes a barrier to full participation (Jerlinder et al. 2009). This may be particularly problematic for adolescents with an ASD.

A better developed and organized interdisciplinary collaboration between educational and health care personnel could give a more comprehensive view of physical inactivity problems and thereby offer solutions to increase PA. Finally, the families of adolescents with an ASD have a vital and proactive role, since they were often reported by the adolescents as the preferred activity partner. This suggests that the families ought to be included in collaboration with stakeholders involved in PA enhancement interventions.

## Conclusions

Adolescents with an ASD express a variety of individual conditions for participation in PA. Different individual conditions, if not recognized, decrease their willingness to be physically active, which negatively affects future PA habits. We propose that knowledge of these individual conditions is a key factor for increasing participation in PA among adolescents with an ASD. The in-depth interviews in this study provide a preliminary understanding of the perspectives of adolescents with an ASD on participation in PA, with implications for understanding their participation in activities in general.
